# Targeted therapies in lung cancer: personalizing treatment across the age spectrum

**DOI:** 10.3389/fonc.2026.1743620

**Published:** 2026-02-25

**Authors:** Yuting Xu, Fei Chen, Honggang Zhang, Xiaofei Wang

**Affiliations:** Department of Ultrasound, Zibo Central Hospital, Zibo, China

**Keywords:** age-related differences, immunotherapy, lung cancer, precision oncology, targeted therapy

## Abstract

Lung cancer remains the leading cause of cancer-related mortality, yet current precision oncology approaches remain overwhelmingly tumor-centric, guided by genomic alterations and immune biomarkers, while largely neglecting the profound impact of aging biology on treatment response. While emerging evidence suggests that aging biology can modify therapeutic benefit and toxicity, its clinical integration remains uneven and largely investigational. In this review, we explicitly distinguish the chronological aging from biological aging to clarify how host biology modifies therapeutic benefit and toxicity. We synthesize mechanistic, translational, and early clinical evidence, while explicitly noting areas where prospective validation is lacking, to reframe personalization of lung cancer therapy through an age-conscious lens. We summarize data indicating that immunosenescence is associated with T-cell exhaustion, myeloid dominance, and extracellular matrix stiffening, features that may contribute to immune-evasive tumor phenotypes and attenuated responses to immune checkpoint blockade in subsets of patients, while pediatric cases, though rare, illustrate how global precision initiatives like iTHER and ZERO enable cautious adaptation of adult therapies. Moving beyond chronological age, we discuss biological age biomarkers, including PhenoAgeAccel, epigenetic clocks, telomere length, and frailty indices, which outperform traditional metrics in predicting risk, resistance, and toxicity, and propose integrating these tools into trial design, screening, and care planning which show promise for risk stratification and toxicity prediction but are not yet validated for routine treatment selection. Looking forward, we outline investigational strategies at the intersection of geroscience and oncology, including immune engineering, senolytics, microenvironmental modulation, and AI-driven multi-omic modeling. Overall, this review argues that biological age represents a critical but still underdeveloped dimension of precision oncology, and highlights key evidence gaps that must be addressed before age-aware personalization can be implemented in routine lung cancer care.

## 1 Introduction

Lung cancer remains the leading cause of cancer-related mortality worldwide, causing approximately 15,000 deaths annually in individuals under 45 years, over 600,000 deaths among those aged 45–64 years, and nearly 1.2 million deaths in people aged 65 years and older, together exceeding 1.8 million deaths per year (1, 2). Despite advances in screening, imaging, and early detection, most cases are still diagnosed at advanced stages, contributing to persistently high lethality. Over the past two decades, however, the therapeutic landscape has shifted dramatically. The identification of oncogenic drivers such as *EGFR, ALK, ROS1, RET, HER2, MET*, and *KRAS*, alongside the clinical success of immune checkpoint inhibitors (ICIs) targeting *PD-1, PD-L1*, and *CTLA-4*, has ushered in an era of precision oncology (3). For biomarker-selected patients, these therapies have achieved survival gains once considered unattainable, underscoring the transformative power of targeted and immune-based approaches.

Yet, these breakthroughs also expose the boundaries of current personalization strategies. Precision oncology in lung cancer remains largely tumor-centric, guided by genomic alterations, PD-L1 expression, or tumor mutational burden. While invaluable, this framework often neglects host-level determinants that critically shape therapeutic efficacy and safety (4–8). Among these, age emerges as one of the most underrecognized, yet biologically decisive, modifiers of cancer therapy. Age influences treatment response through diverse biological and clinical mechanisms, spanning pharmacokinetics, immune competence, comorbidity burden, and survivorship considerations. These age-related dynamics help explain why outcomes diverge so markedly across the lifespan and why frameworks that treat age merely as a chronological cutoff fall short of true personalization (9–13). Importantly, “age” denotes chronological time, whereas “aging” refers to biological processes, including immunosenescence, inflammaging, and tissue/cellular senescence, that reprogram host defenses and thereby alter lung cancer behavior and treatment outcomes. These distinctions help explain why patients with similar tumors may experience markedly different responses across the lifespan and why reliance on chronological age alone falls short of true personalization. At the same time, it remains uncertain which aging-related features are sufficiently robust to guide real-world treatment decisions, underscoring the need for cautious interpretation of existing data.

Against this backdrop, a central challenge emerges, which is how to reconcile tumor-centric precision oncology with the biological heterogeneity introduced by aging. Rather than treating age as a statistical covariate or eligibility criterion, it may need to be considered an integral dimension of personalized care. In this review, we examine how age intersects with targeted therapies and immunotherapies in lung cancer across pediatric, adult, and elderly populations, highlighting current gaps in trial design, biomarker development, and clinical evidence. We further discuss emerging strategies to integrate age-conscious principles into translational research and clinical practice, to advance more biologically informed, equitable, and durable precision oncology across the lifespan.

## 2 Rethinking age in precision oncology

The prevailing framework of precision oncology in lung cancer is predominantly tumor-centered, anchored in actionable genomic alterations and immune biomarkers such as PD-L1 expression or tumor mutational burden. While this paradigm has yielded transformative therapies, it underestimates the profound influence of host biology on therapeutic efficacy and safety. Among host-related variables, age represents one of the most underappreciated and complex modifiers of cancer therapy. It is not merely a demographic characteristic but a biological determinant that shapes pharmacokinetics, immune dynamics, comorbidity burden, and survivorship.

Chronological age is an imprecise proxy for fitness, while biological age captures cumulative functional decline across tissues and immune compartments, explaining why patients of the same chronological age can have divergent pharmacokinetics, toxicity profiles, and responses to targeted therapy and immunotherapy (14). A key limitation in current practice is the reliance on chronological age as a surrogate for patient fitness. Clinical trial eligibility and treatment algorithms frequently apply arbitrary cutoffs (e.g., ≥65 or ≥70 years, or defining “early-onset” as <50 years), yet these thresholds fail to capture biological heterogeneity (15). Evidence from large cohort studies highlights this limitation. In one analysis of more than 25,000 cancer patients, using 50 years as a universal cutoff for hereditary cancer testing would have failed to detect nearly 400 clinically significant germline mutations (16). Likewise, patients with early-onset cancers frequently carry a higher burden of pathogenic germline variants, emphasizing that cancer risk and treatment decisions should be guided by tumor biology rather than arbitrary age thresholds (17). From a molecular standpoint, precision oncology is fundamentally “age-agnostic” (18). Genomics-driven approaches have revealed actionable alterations across the lifespan, from pediatric to geriatric patients. Tumor-agnostic approvals for alterations such as *NTRK fusions* and high microsatellite instability exemplify how targeted therapies transcend age categories (19). Large-scale efforts like the GENIE Consortium further demonstrate that ~30% of tumors harbor clinically actionable drivers in both younger and older patients, with sequencing-matched therapy consistently improving outcomes regardless of age (20). These data reinforce the principle that tumor biology, not patient age, should guide therapeutic choice.

Despite this, structural age bias persists. Older adults comprise more than half of all cancer cases, yet account for only about one-third of clinical trial participants. Protocols often exclude patients with comorbidities or organ dysfunction, disproportionately limiting access for those over 65 and especially over 75. This underrepresentation hampers accurate assessment of dosing, safety, and efficacy in the very populations most affected by lung cancer (21, 22). Recent regulatory and research initiatives call for greater inclusion of elderly patients in trials, with adapted designs that account for frailty, organ reserve, and polypharmacy rather than blanket exclusions.

Looking ahead, a redefinition of age in precision oncology is urgently needed. Instead of relying on fixed chronological cutoffs, age classification should be specific to tumor type and incorporate molecular and germline testing strategies to better identify high-risk or targetable subgroups. Advances in biological age biomarkers, such as epigenetic clocks, immune repertoire profiling, and senescence-associated signatures, provide tools to improve stratification and customize therapy beyond basic demographics. Multi-omics and longitudinal studies are beginning to reveal how cumulative mutational load, immunosenescence, and metabolic reprogramming influence therapeutic vulnerabilities across the lifespan. In this context, rethinking age is not about abandoning demographic categories but about incorporating functional, molecular, and geroscience-based measures of aging into precision oncology. This transition promises more equitable and effective personalization, ensuring therapies are optimized not only for tumors but also for the patients who have them. Ultimately, since most lung cancers occur after the sixth decade, any attempt to adjust standards of care based on age must be grounded in age- and stage-matched evidence rather than demographics alone, a limitation we will address in the following sections.

## 3 Targeted therapies through the age spectrum in lung cancers

Targeted therapies have transformed cancer treatment by enabling personalized interventions based on defined molecular drivers. These strategies now extend across the lifespan, from pediatric to elderly populations, yet their efficacy, safety, and application vary according to tumor biology, comorbidities, and age-related physiology.

### 3.1 Pediatric lung cancers and targeted therapies

Primary lung cancer in children is exceptionally rare, accounting for fewer than 0.5% of pediatric malignancies (23). Most pediatric lung tumors are metastatic lesions from sarcomas or other primary cancers rather than *de novo* lung carcinomas, and when primary lung cancers do occur, they are typically bronchial carcinoid tumors, pulmonary blastomas, or very rare pediatric presentations of non-small cell lung cancer (NSCLC) (24). This rarity has historically limited the development of pediatric-specific therapeutic strategies, resulting in a strong reliance on agents first tested and approved in adults. Increasingly, however, advances in molecular diagnostics and next-generation sequencing (NGS) are enabling a precision medicine approach, in which treatment is guided by tumor biology rather than patient age, allowing children to access therapies originally designed for adult lung cancer.

Evidence shows that pediatric lung cancers harbor actionable alterations similar to those in adults, most commonly involving ALK, ROS1, EGFR, BRAF, KRAS, RET, and NTRK genes (25). Targeted therapies that inhibit these drivers have demonstrated striking efficacy in select pediatric cases. For example, crizotinib and lorlatinib achieve high response rates in *ALK*-driven tumors, with lorlatinib offering enhanced central nervous system penetration and activity against resistance mutations (26). Similarly, osimertinib, gefitinib, and erlotinib have proven effective in pediatric patients with *EGFR* mutations, while crizotinib is used in *ROS1*-rearranged tumors (27). The dabrafenib-trametinib combination, already approved for *BRAF V600E*-mutated pediatric glioma, has been extended to rare lung cancers with the same mutation (28). In addition, selpercatinib and larotrectinib have demonstrated robust activity against *RET* and *NTRK* fusions, respectively, and are approved in a tissue-agnostic setting, further supporting their adaptation for rare pediatric lung cancers (29).

Because large-scale randomized trials are not feasible for such rare diseases, pediatric lung cancer management relies heavily on basket trials (30), precision medicine initiatives, and rare tumor registries. Initiatives like the ZERO Childhood Cancer Program in Australia (31) and the pediatric arms of NCI-MATCH have shown that NGS-driven treatment recommendations can improve outcomes in high-risk pediatric cancers (32). The iTHER (Individualized Therapies for Children with Cancer) program, a European precision oncology initiative, is an important example of how sequencing and multidisciplinary tumor boards can be successfully integrated into clinical practice. iTHER has demonstrated that actionable genomic alterations can be identified in the majority of children with relapsed or refractory cancers, including rare lung tumors, and that these findings often inform treatment decisions, trial enrollment, or compassionate use of novel agents (33). Such programs represent critical infrastructure for translating molecular insights into personalized care, especially in ultra-rare cancers such as pediatric lung malignancies.

Despite these successes, pediatric lung cancer remains constrained by major evidence gaps, because cases are ultra-rare and treatment is frequently extrapolated from adult oncology. However, translating adult protocols to children is neither pharmacologically nor biologically straightforward, and limited global access to comprehensive molecular profiling further compounds this problem. Moreover, when actionable alterations are identified, pediatric dosing guidance, formulations, and drug availability can lag behind adult practice. In our understanding, “borrowing” adult-targeted therapy regimens for pediatric patients can introduce uncertainty in both exposure and risk. Developmental pharmacokinetics/pharmacodynamics can alter absorption, hepatic metabolism, renal clearance, and target engagement, so simple body-size-based dose scaling cannot replace pediatric dose-finding. In parallel, the difference in toxicity profiles of children can lead to acute effects and late sequelae like endocrine and growth disruption, bone health impairment, neurocognitive effects, fertility risks, and cumulative consequences of long-term on-target/off-target inhibition during development, which are rarely measured in adult trials (34). Further, efficacy can also diverge, as pediatric primary lung tumors and pediatric lung metastases treated with lung-cancer agents may have distinct lineage programs and immune contexts compared with smoking-associated adult NSCLC, thus potentially reshaping both resistance trajectories and durability of response (24). Collectively, these factors justify pediatric-specific phase I/II studies, standardized long-term follow-up, and registry-based pharmacovigilance whenever adult agents are repurposed for childhood lung cancers. Moving forward, the management of pediatric lung cancers will depend on international collaboration, comprehensive molecular testing, and the expansion of adaptive trial platforms. Incorporating programs like iTHER, ZERO, and NCI-MATCH into routine practice worldwide, alongside broader participation in global registries and rare tumor networks, will be essential to close the knowledge gap for these ultra-rare cancers. Emerging therapies, including next-generation *KRAS* inhibitors and bispecific T-cell engagers like tarlatamab, currently under study in adult lung cancer (35), offer promising avenues for pediatric adaptation based on molecular drivers rather than age. Together, these advances position pediatric lung cancer care firmly within the broader precision oncology revolution, ensuring that even the rarest cancer populations benefit from biology-driven, individualized treatment.

### 3.2 Targeted therapy in adults and older lung cancer patients

Targeted therapies have transformed the treatment landscape of adult and older patients with lung cancer, providing highly effective, precision-guided alternatives to chemotherapy. Advances in molecular diagnostics have identified actionable alterations in both NSCLC and SCLC, leading to the development of tyrosine kinase inhibitors (TKIs), antibody-drug conjugates (ADCs), and bispecific immunotherapies that are now central to care (36).

In NSCLC, TKIs remain the foundation of targeted therapy. EGFR inhibitors such as osimertinib, gefitinib, and erlotinib are standard of care, with osimertinib widely used for both metastatic disease and as adjuvant therapy to prevent recurrence (37). Osimertinib’s favorable safety profile and CNS activity make it particularly suitable for older patients (38), while erlotinib and gefitinib remain effective, well-tolerated options that provide meaningful symptom control. ALK inhibitors (crizotinib, alectinib, ceritinib, lorlatinib) achieve durable disease control and intracranial efficacy (39), and ROS1 inhibitors (crizotinib, entrectinib, taletrectinib) extend precision therapy to ROS1-positive patients, including those with resistance mutations (40). Other targeted approaches include BRAF/MEK inhibitors (dabrafenib, trametinib) (41), MET inhibitors (capmatinib, tepotinib) (42), and RET and NTRK inhibitors (selpercatinib, pralsetinib, larotrectinib) (43). A major milestone has been the approval of KRAS G12C inhibitors, sotorasib and adagrasib, which provide effective treatment for a mutation long considered undruggable (44).

In SCLC, targeted therapies are emerging, most notably tarlatamab (Imdelltra^®^), a bispecific T-cell engager approved in 2024 for recurrent SCLC, which demonstrated a 40% reduction in mortality compared to chemotherapy with a more favorable safety profile (45). Novel ADCs targeting c-MET, TROP2, and EGFR exon20 insertions are also in development, and combination regimens, such as EGFR inhibitors with MET inhibitors (osimertinib plus savolitinib), are addressing acquired resistance in NSCLC (46). Recent FDA approvals in 2025 have further expanded precision options, particularly benefiting older adults, for whom aggressive chemotherapy is often poorly tolerated. Datopotamab deruxtecan (Datroway), approved for EGFR-mutant NSCLC after EGFR therapy and chemotherapy, introduces a new ADC option for heavily pretreated patients (47). Zenocutuzumab (Bizengri) offers a targeted approach for NRG1 fusion-positive NSCLC (48), while taletrectinib (Ibtrozi) extends therapy for ROS1-positive patients with resistance mutations (49). The oral HER2-targeted therapy HERNEXEOS^®^**^51^** and the lazertinib-amivantamab combination offer additional targeted approaches (50), reducing chemotherapy dependence and hospital visits while improving quality of life, critical considerations for elderly care. Targeted therapies have proven efficacy in older populations, including those over 70 years of age, with agents such as osimertinib, erlotinib, gefitinib, sotorasib, and adagrasib demonstrating strong disease control with manageable toxicity (51). Bevacizumab remains an option in non-squamous NSCLC but requires careful risk-benefit evaluation due to increased cardiovascular and bleeding risks in elderly patients (52). In frail or comorbid patients, targeted monotherapy is generally preferred to maximize tolerability, while ALK and ROS1 inhibitors remain highly effective but are supported by limited elderly-specific data compared to EGFR and KRAS-directed drugs (53).

Overall, targeted therapy efficacy is largely age-independent, but safe implementation in older adults requires thoughtful dose adjustments, drug-drug interaction management, and incorporation of geriatric oncology principles. The expansion of oral, outpatient-based therapies and the increasing availability of ADCs and bispecific antibodies are particularly advantageous for elderly patients, improving convenience and preserving independence. As trial representation broadens and real-world data collection grows, age-conscious precision oncology will ensure that older adults receive the same therapeutic innovations driving lung cancer survival gains in younger populations. A concise summary of current targeted therapy classes, molecular drivers, key agents, and geriatric-specific considerations is presented in **Table 1**, highlighting practical adaptations required to ensure safe and effective use of precision therapies across the aging population.

**Table 1:** Targeted therapy landscape in lung cancer: molecular drivers, representative agents, and geriatric-adaptive considerations for precision treatment.

Therapy class (mechanisms)	Actionable biomarkers	Flagship agents	Geriatric-priority considerations	Practical perals
EGFR TKIs (36)	EGFR activating mutations (L858R, uncommon: G719X, S768I, L861Q)	Osimertinib, Erlotinib, Gefitinib; ± Lazertinib + Amivantamab	ILD risk, rash/diarrhea → dehydration; hepatic/renal monitoring; polypharmacy (CYP3A)	Osimertinib has strong CNS penetration; adjuvant use reduces recurrence; dose interruptions safer than empiric reductions in frail pts
ALK inhibitors (39)	ALK fusions	Alectinib, Lorlatinib, Crizotinib	Dyslipidemia, neurocognitive/neuropathy (esp. lorlatinib); CK↑, hepatic labs	Excellent intracranial control; prefer alectinib first-line in older adults for tolerability
ROS1 inhibitors (26, 27)	ROS1 fusions	Entrectinib, Crizotinib	QTc prolongation, edema; DDIs (CYP3A)	Entrectinib has CNS activity; taletrectinib active in resistance settings
KRAS G12C inhibitors (35, 44, 51)	KRAS p.G12C	Sotorasib, Adagrasib	Hepatotoxicity; GI effects; DDIs with acid-reducing agents	Oral, generally well tolerated in ≥70; watch for cumulative toxicity with combinations
RET inhibitors (43)	RET fusions	Selpercatinib, Pralsetinib	Hypertension, LFT abnormalities; QTc	Durable responses across ages; monitor BP closely in vascular comorbidity
MET inhibitors (42)	MET exon14 skipping, MET amp	Capmatinib, Tepotinib	Peripheral edema (falls risk), ILD; renal function	Useful in older, smoking-associated NSCLC; edema management critical
BRAF/MEK combo (28, 41)	BRAF V600E	Dabrafenib, Trametinib	Pyrexia, fatigue→ deconditioning; cardiomyopathy/ocular events	Dose holds/stepwise re-challenge prevent treatment abandonment
HER-2 targeted (47, 54)	HER2 mutations/overexpression	Trastuzumab deruxtecan; TKIs like Zongertinib	ILD/pneumonitis risk higher in older pts; nausea, cytopenias	Early imaging + pulse ox if dyspneic; lower threshold to hold for ILD work-up
NTRK Inhibitors (29, 40, 43)	NTRK fusions	Larotrectinib, Entrectinib	Dizziness, transaminases; DDIs	Tissue-agnostic; rapid, durable responses even in frail patients
VEGF/VEGFR inhibitors (52, 55)	VEGF pathway	Bevacizumab, Ramucirumab	Hypertension, bleeding, thromboembolism, wound-healing	Avoid with recent hemoptysis/CVD; ambulatory BP monitoring helpful
Immune checkpoint inhibitors (3, 11, 56)	PD-1/PD-L1, CTLA-4	Pembrolizumab, nivolumab, ipilimumab	Immune AEs may be harder to detect/manage in multimorbidity; possible attenuated efficacy with immunosenescence	Remain effective in many ≥75; baseline thyroid/HbA1c; plan for rapid steroid access
ADCs/Bispecifics (45, 47)	DLL3 (SCLC), HER2, TROP2, EGFRex20	Datopotamab-deruxtecan, tarlatamab (SCLC), trastuzumab deruxtecan	Stomatitis, cytopenias, ILD (ADCs); CRS/neurologic events (bispecifics)	Chemo-sparing options suited to frail pts; pre-empt oral care and ILD surveillance

Although targeted therapies are designed to be tumor-genotype-driven and are often described as “age-independent,” treatment outcomes across the lifespan remain shaped by age-associated host biology. In older patients, immunosenescence and chronic low-grade inflammation/inflammaging can indirectly influence targeted-therapy benefit by shifting baseline tumor-immune setpoints, remodeling the TME, and increasing vulnerability to complications like infections, pneumonitis/ILD, and functional decline (57). These host factors become especially decisive when targeted therapy is combined with, or followed by, immune checkpoint inhibitors, where effective antitumor immunity contributes to durable control. Accordingly, a mechanistic understanding of immune aging is essential to explain why tumors with the same actionable driver can display different depth and durability of response, and different toxicity trajectories, across age groups, particularly in biologically older adults.

## 4 Effects of immune aging on immunotherapy for lung cancers

**Figure 1:**
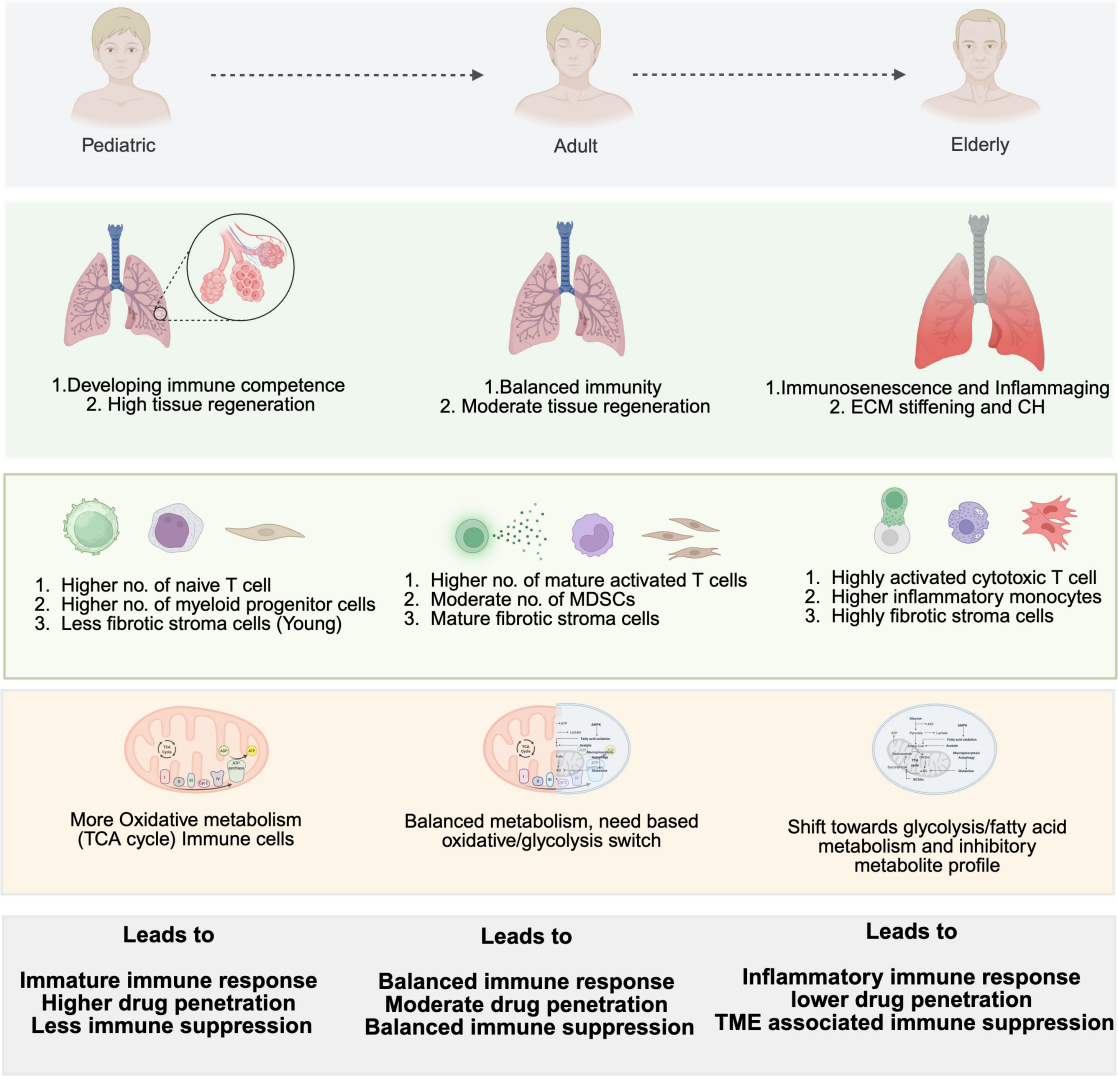
Age-associated remodeling of immune, stromal, and metabolic landscapes shaping lung cancer therapy response. Schematic representation of how aging reprograms the immune system, stroma, and metabolism across the lifespan, from pediatric to elderly individuals, thereby influencing lung cancer biology and therapeutic outcomes. Pediatric lungs exhibit developing immune competence, high regenerative capacity, and oxidative immune metabolism with minimal fibrosis, resulting in higher drug penetration and low immune suppression. Adults maintain balanced immunity and moderate stromal maturity, allowing stable drug response and moderate immune regulation. In contrast, elderly lungs display immunosenescence, inflammaging, extracellular matrix (ECM) stiffening, and clonal hematopoiesis (CH), with predominance of inflammatory monocytes, fibrotic stroma, and glycolytic/fatty acid-biased metabolism. These changes collectively promote immune exhaustion, reduced drug penetration, and therapy resistance within the tumor microenvironment.

Immune aging, also termed immunosenescence, is an age-associated immune remodeling that is not merely immune “decline” but a systemic reprogramming of innate and adaptive immunity characterized by reduced naïve lymphocyte production and repertoire diversity, accumulation of dysfunctional/exhausted effector subsets, myeloid skewing, and a chronically inflamed baseline state called “inflammaging**”** (58). These age-linked changes profoundly affect lung cancer immunotherapy by altering systemic immune competence and reshaping the TME, thereby modifying both the efficacy and toxicity of ICIs. Importantly, these effects are heterogeneous across patients and tumor contexts, and not all older individuals exhibit clinically meaningful immunosenescence. Rather than a uniform loss of function, immune aging is dynamic and heterogeneous, involving chronic inflammatory signaling, immune exhaustion, myeloid dominance, and stromal remodeling processes that collectively determine whether an antitumor response can be initiated and sustained (58). An overview of age-associated changes in immune, stromal, and metabolic landscapes shaping lung cancer biology and therapy response is illustrated in **Figure 1**. Understanding these mechanisms is key to optimizing treatment for the growing population of older adults with lung cancer, who remain underrepresented in clinical trials but comprise the majority of cases.

### 4.1 Mechanistic drivers of immunosenescence-associated lung cancer

Cellular senescence, defined by permanent growth arrest and SASP, is distinct from immunosenescence, the age-related weakening of innate and adaptive immunity. Along with systemic inflammaging, these processes help explain how host aging undermines antitumor immune responses in lung cancer. Immunosenescence-associated lung cancer emerges from a complex interplay of chronic inflammation, T-cell dysfunction, TME remodeling, and dysregulation of cell death and metabolic pathways (59). Aging fundamentally alters both adaptive and innate immunity, reshaping the immune landscape in ways that promote tumor growth and resistance to immunotherapy. One of the earliest hallmarks of immune aging is thymic involution, which sharply reduces the output of naïve T cells and narrows the TCR repertoire (60). This limits the immune system’s capacity to recognize the diverse neoantigens characteristic of NSCLC (61). Senescent CD8^+^ T cells, which accumulate with age, exhibit diminished cytotoxicity, impaired proliferation, and elevated expression of inhibitory receptors such as PD-1, TIM-3, LAG-3, and CTLA-4. Clinical data in elderly NSCLC patients demonstrate that high frequencies of these exhausted T-cell subsets correlate with reduced responsiveness to PD-1 blockade and shorter progression-free survival, underscoring the clinical relevance of T-cell senescence (62). Notably, most supporting evidence for these associations derives from correlative clinical studies and translational analyses rather than prospective biomarker-guided trials, and causality has not been definitively established.

Senescent T cells not only lose effector function but also contribute to a pro-tumorigenic environment through the SASP, releasing cytokines such as IL-6, IL-8, and TNF-α, which drive chronic inflammation, angiogenesis, and tumor progression (63). This inflammation creates a persistent low-grade inflammatory state supported by factors like MCP-1 and CRP, enhancing immune suppression and metastatic potential (11). Senescent T cells also exhibit reduced expression of costimulatory molecules such as CD28 and increased levels of apoptotic markers like CD95, making them prone to apoptosis through Fas/FasL interactions. This selective depletion of functional T cells, combined with tumor cell evasion of apoptotic signals, further impairs immune surveillance and promotes tumor escape (64). The aging immune system also undergoes metabolic and signaling alterations that exacerbate dysfunction. Chronic metabolic stress and nutrient competition within the TME activate pathways such as AMPK, p38 MAPK, and ATM, inducing T-cell cycle arrest through upregulation of p16, p21, and p53 (65). Mitochondrial dysfunction in aged T cells elevates ROS levels, which drive DNA damage, impair effector functions, and accelerate senescence. These intracellular changes are amplified by tumor-derived factors that manipulate immune signaling through cAMP/PKA-CREB, NF-κB, and cGAS-STING pathways, further suppressing T-cell activation and skewing immune cell metabolism toward glycolysis, reducing the durability of antitumor immunity (66, 67).

Similarly, parallel changes in innate immunity reinforce immunosuppression. Aging hematopoietic stem cells exhibit a myeloid differentiation bias, leading to expansion of immunosuppressive populations such as MDSCs and M2-like macrophages. These cells secrete TGF-β, IL-10, VEGF, and arginase-1, dampening antigen presentation and cytotoxic T-cell infiltration (68). Preclinical lung adenocarcinoma studies have shown that age-associated IL-1α/β-driven myelopoiesis accelerates tumor growth and immune evasion, revealing systemic inflammation as a central driver of lung cancer aggressiveness in older individuals (69). Moreover, the checkpoint signaling landscape shifts with age, further compounding resistance to ICIs. NSCLC tumors from elderly patients often display elevated PD-L1 and PD-L2 expression, and stromal cells within aged TMEs upregulate additional inhibitory ligands such as Galectin-9 and B7-H4 (56). This expanded network of checkpoint signaling diminishes T-cell reinvigoration even in tumors classified as PD-L1-high, highlighting a fundamental disconnect between biomarker predictions and therapeutic outcomes in older patients (70). Together, these adaptive and innate immune alterations transform the lung cancer TME into an immunologically “cold” state that favors tumor progression and immune escape.

Collectively, immunosenescence in lung cancer reflects more than just chronological age, rather it is a biologically driven state defined by T-cell exhaustion, chronic inflammation, myeloid dominance, and extensive metabolic reprogramming. These changes blunt the efficacy of ICIs and other immunotherapies, explaining why older patients often experience lower response rates despite harboring tumors with actionable mutations or high PD-L1 expression. Addressing these challenges will require therapeutic approaches that restore T-cell diversity, neutralize myeloid suppression, and target inflammatory pathways to remodel the TME. By integrating these mechanistic insights into biomarker development and treatment design, precision immunotherapy can evolve beyond tumor-centric models to overcome the profound influence of immune aging in lung cancer.

### 4.2 Inflammaging and tumor microenvironment remodeling in lung cancer

Aging profoundly reshapes the lung’s immune and stromal landscape, fostering a pro-tumorigenic microenvironment that accelerates both cancer initiation and progression. Chronic, low-grade inflammation, or inflammaging, is a hallmark of this process, driven by the persistent secretion of cytokines, chemokines, and growth factors from senescent immune and stromal cells. This SASP is characterized by elevated IL-6, IL-8, TNF-α, VEGF, and CRP, all of which disrupt local tissue homeostasis, promote angiogenesis, and create a permissive niche for malignant transformation (71). In NSCLC, elevated baseline IL-6 and CRP levels have been correlated with inferior responses to PD-1 inhibitors, illustrating how systemic inflammation directly undermines immunotherapy efficacy in older patients (72).

At the cellular level, aging lungs exhibit heightened activation of inflammatory pathways, including NFκB, p38 MAPK, and interferon-gamma signaling, alongside increased NLRP3 inflammasome activity (73). NLRP3-driven production of IL-1β and IL-18 fuels tumor-promoting inflammation, while chronic antigenic exposure and epigenetic drift erode immune surveillance, further impairing responses to checkpoint blockade (74). These immune changes are compounded by structural remodeling of the lung’s extracellular matrix (ECM), which intensifies with age. Increased deposition and crosslinking of collagen I, accompanied by reduced elastin and laminins, stiffens lung tissue and reduces elasticity, compromising epithelial integrity and amplifying oncogenic signaling. Elevated fibronectin levels further enhance growth factor presentation, supporting tumor cell adhesion and proliferation even before overt malignancy emerges (75, 76). Mechanical alterations to the ECM profoundly influence intracellular signaling cascades. Stiffened matrices activate integrin-FAK signaling, leading to downstream activation of YAP/TAZ, β-catenin, and ERK pathways, all of which drive proliferation, stem-like cell behavior, and therapy resistance (77). These changes also trigger the differentiation of fibroblasts into myofibroblasts via TGF-β and PDGF, creating a vicious cycle of fibrosis and ECM deposition that reinforces immune exclusion (78). Such changes contribute to the “immune desert” phenotype frequently observed in older NSCLC patients, where cytotoxic T-cell infiltration is physically restricted, and immune checkpoints are upregulated on both tumor and stromal cells (79).

Together, inflammaging and ECM remodeling converge to create a tumor microenvironment that is inflamed yet immunosuppressed, structurally rigid, and primed for malignant progression. Transcriptomic and histopathological analyses reveal that ECM signatures in aged lungs closely resemble those observed in high-risk, aggressive NSCLC subtypes, directly linking aging biology to worse clinical outcomes. While emerging interventions, including NLRP3 inflammasome inhibitors, TGF-β-targeted therapies, integrin antagonists, and senolytics, offer promise in reversing some features of inflammaging and stromal fibrosis, these strategies remain largely investigational (80). Addressing these age-specific microenvironmental changes will be essential to improving the efficacy of immunotherapies and targeted therapies in elderly lung cancer patients, who currently face disproportionate resistance and relapse rates.

### 4.3 Clonal hematopoiesis and age-dependent comorbidities in lung cancer

Age rarely acts alone, as it interacts with comorbid biological processes that shape both therapeutic efficacy and toxicity. Among these, clonal hematopoiesis (CH), which is an age-associated expansion of hematopoietic progenitor clones carrying somatic mutations in genes such as *DNMT3A, TET2, ASXL1, JAK2*, and *TP53*, has emerged as a critical determinant of systemic inflammation and immune dysregulation (81). CH-derived myeloid cells exhibit a heightened pro-inflammatory phenotype marked by excess IL-1β and IL-6 secretion, oxidative stress, and skewed emergency myelopoiesis, all of which reinforce inflammaging, promote M2-like macrophage polarization, and contribute to immune exclusion within the TME (82). In solid tumors, including NSCLC, CH has been associated with distinct immune profiles characterized by an increased fraction of exhausted CD8^+^ T cells, higher burden of cardio-pulmonary comorbidity, and greater risk of treatment-related toxicity such as radiation pneumonitis and immune checkpoint inhibitor-related myocarditis (83). It may also complicate molecular diagnostics when hematopoietic variants are misinterpreted as tumor-derived mutations in liquid biopsies. Although lung cancer-specific studies remain limited, converging evidence supports the view that hematopoietic aging and CH fuel systemic inflammation and myeloid dominance, thereby diminishing ICI responsiveness and elevating toxicity risk (84). For older or multimorbid patients, particularly those with COPD, cardiovascular disease, or elevated inflammatory markers such as CRP, incorporating CH screening and cytokine profiling into clinical evaluation may refine biological age assessment, improve risk prediction, and guide treatment intensity. Integrating CH status alongside PD-L1 expression, tumor mutational burden, and frailty indices into trial design represents a practical step toward a more comprehensive, age-aware model of precision immuno-oncology.

## 5 Biological age biomarkers and predictive tools for lung cancer therapy

Chronological age has long been used as a proxy for fitness in lung cancer management, yet it fails to capture the wide heterogeneity of aging biology, particularly in predicting therapy tolerance, response, and long-term outcomes. Recent advances in biological age biomarkers offer a more precise understanding of patient vulnerability, integrating genetic, environmental, metabolic, and immune factors that collectively shape cancer risk and therapeutic resilience. These tools are emerging as a cornerstone of age-conscious oncology, allowing for treatment planning that reflects physiological rather than chronological age.

Among the most promising measures is PhenoAgeAccel, a composite index derived from standard clinical blood biomarkers, including CRP, glucose, albumin, alkaline phosphatase, and blood cell counts (85). Large cohort studies, such as those from the UK Biobank, demonstrate that PhenoAgeAccel is independently associated with increased lung cancer risk, even after adjusting for polygenic risk scores (PRS) and chronological age (86). Individuals classified as biologically older by PhenoAgeAccel display significantly higher five-year absolute risk of lung cancer than their chronologically matched peers, while Mendelian randomization studies suggest a causal link between accelerated biological aging and lung cancer susceptibility. Importantly, combining PhenoAgeAccel with PRS improves risk stratification, offering additive predictive power that reclassifies some individuals into higher or lower risk categories than genetic data alone would indicate. This integration has significant implications for refining screening eligibility and early detection strategies, potentially enabling earlier interventions for biologically older patients who would otherwise be overlooked by PRS-based models. Other biomarkers of biological aging are equally promising (87). Epigenetic clocks, including DNA methylation age and GrimAge, estimate biological age from methylation patterns that integrate cumulative exposures and cellular stress. GrimAge further incorporates smoking and protein-related methylation signals, allowing it to outperform chronological age in predicting lung cancer risk and therapy outcomes (88). Shortened leukocyte telomere length, which reflects cumulative replicative stress and cellular aging, is another established marker of accelerated aging and has been associated with poorer outcomes and increased lung cancer risk, further supporting its role in prognostication (89). Circulating inflammatory markers such as IL-6, TNF-α, CRP, and p16^INK4a^ capture systemic inflammaging, immune senescence, and therapy vulnerability. It correlates with frailty, reduced ICI responsiveness, and higher complication rates (90). Organ-specific measures like “lung age,” calculated from spirometry indices such as FEV1, capture pulmonary reserve and are associated with postoperative risk and treatment tolerance. When integrated with systemic biological age scores, pulmonary metrics contribute to more accurate risk models for surgical and treatment planning (91). Similarly, comprehensive geriatric assessments and frailty indices combine clinical measures of functional reserve with emerging molecular biomarkers to guide therapy personalization in elderly and comorbid patients (92). A summary of key biological aging biomarkers with mechanistic underpinnings and implications for lung cancer precision therapy is presented in **Table 2**.

**Table 2:** Emerging biomarkers of biological aging and their clinical relevance for lung cancer precision therapy.

Biomarker/Composite	Mechanistic basis	Clinical implications in lung cancer precision therapy
PhenoAge/PhenoAgeAccel (85, 86)	Composite index derived from routine clinical biomarkers (e.g., CRP, glucose, albumin, alkaline phosphatase, blood cell counts) capturing multisystem physiological aging	Strong predictor of lung cancer risk, outcomes, and mortality independent of chronological age; integrates with polygenic risk scores to enhance screening and stratification accuracy.
DNA Methylation Age (Epigenetic Clocks) (88)	Genome-wide DNA methylation patterns reflecting cumulative epigenetic drift (e.g., Horvath, Hannum, GrimAge)	Accelerated epigenetic age associates with higher lung cancer risk and poorer prognosis; enables biological-age–based patient stratification and potential monitoring of anti-aging interventions.
Leukocyte Telomere Length (LTL) (89)	Shortening of telomeres in peripheral leukocytes indicating replicative and oxidative stress–related aging	Reduced LTL correlates with increased lung cancer susceptibility and adverse outcomes; may serve as a component of integrated predictive or therapeutic-response models.
Inflammatory and Senescence Markers (e.g., IL-6, CRP, TNF-α, p16^INK4a^) (63, 71)	Chronic systemic inflammation and SASP driving immune dysfunction and tumor-promoting microenvironment	Elevated inflammatory markers denote accelerated biological aging, diminished immunotherapy efficacy, and increased toxicity risk; inform anti-inflammatory or immune-supportive adjunct strategies.
Functional/Physiological Aging Indices (91, 92)	Composite measures such as frailty index, comprehensive geriatric assessment (CGA), and spirometry-based “lung age” capturing organ reserve and resilience	Provide practical estimation of biological age for tailoring therapy intensity, predicting tolerance, and guiding supportive care in older or comorbid patients.

Collectively, these tools shift precision oncology toward biological age-guided care, enabling treatment decisions based on physiological resilience rather than chronological age, with potential benefits for screening, therapy sequencing, and toxicity reduction. While large-scale prospective trials are needed, current evidence strongly supports a future where biological age biomarkers guide precision medicine, ensuring that patients across the age spectrum, from rare pediatric cases to the very elderly, receive appropriately tailored, effective, and less toxic care. Despite growing interest, biological aging biomarkers remain outside current lung cancer guidelines due to limited prospective validation, lack of standardized thresholds, cohort heterogeneity, and uncertain clinical integration. At present, they are best applied for research and risk stratification rather than treatment selection.

## 6 Emerging strategies to reprogram lung cancer therapy across the age spectrum

Lung cancer therapy is evolving beyond tumor genomics toward integrative models that incorporate host aging biology. While many such approaches remain investigational, the convergence of geroscience, precision oncology, and artificial intelligence is enabling more adaptive, patient-centered strategies that account for immune aging, tumor microenvironment dynamics, and physiological resilience.

A major frontier in this evolution is immune rejuvenation, with emerging technologies aiming to reverse the effects of immune senescence rather than bypass them (93). CRISPR/Cas9-based genome engineering enables precise enhancement of T and natural killer cells to improve tumor recognition, checkpoint resistance, and cytokine expression. These advances have accelerated the development of CAR-T and CAR-NK therapies capable of functioning within the immunosuppressive microenvironments of aged lungs (94). iPSC-derived NK and T cells represent another breakthrough, offering robust cytotoxicity, improved persistence, and reduced toxicity compared with traditional donor-derived therapies. These engineered cells are not only highly effective in preclinical lung cancer models but also demonstrate the ability to sensitize resistant tumors to PD-1 inhibitors by promoting T-cell infiltration, providing a potent combination approach for elderly or immunocompromised patients (95). Clinical trials are now validating the safety and efficacy of iPSC-based therapies in advanced lung cancer, offering scalable, standardized solutions that overcome the limitations of aging immune systems.

Innovations in cellular reprogramming are also extending into *in vivo* applications, with strategies to rejuvenate exhausted immune cells directly within the patient. Partial cellular reprogramming through transient expression of Yamanaka factors and targeted epigenetic editing shows promise in resetting senescence-associated dysfunction without inducing full pluripotency, restoring immune function without increasing tumorigenic risk (96). In parallel, senolytic therapies that selectively clear senescent immune and stromal cells, as well as drugs targeting inflammatory circuits like the NLRP3 inflammasome or IL-1 signaling, are under investigation to dismantle the chronic inflammatory feedback loops that blunt immunotherapy responses in older patients (97). Together, these advances aim to create a new therapeutic paradigm where the immune system is actively rebuilt to sustain durable anti-tumor immunity. Remodeling the TME is another crucial pillar of next-generation strategies. Age-related changes in lung tissue architecture, such as ECM stiffening and fibroblast activation, create physical and biochemical barriers that exclude immune cells and limit drug penetration. Novel therapeutics targeting LOX-mediated collagen crosslinking, integrin signaling, and fibroblast activation protein (FAP) are being developed to soften these fibrotic barriers and reprogram the TME into an immune-permissive state (98). Combination approaches pairing these ECM-targeting therapies with immune checkpoint blockade hold particular promise for elderly patients, whose tumors are often characterized by fibrosis, immune exclusion, and resistance to standard immunotherapies. Oncolytic viruses, *in situ* vaccines, and bispecific antibodies are also being tested to ignite immune responses in these otherwise “cold” tumors, demonstrating that therapy can be designed not only to target cancer cells but also to reshape the ecosystem in which they thrive (99).

The integration of AI and multi-omics is accelerating these advances by enabling more precise patient stratification and therapy design. Multi-layered datasets, including single-cell transcriptomics, spatial proteomics, metabolomics, and epigenomic profiling, are being analyzed through AI-driven algorithms to predict resistance mechanisms, toxicity risk, and optimal treatment sequencing. These models are evolving into patient “digital twins,” virtual simulations that allow clinicians to test therapeutic strategies in silico before implementation (100). A clinically useful digital twin is not a static risk score but a continuously updated, patient-specific computational model that integrates tumor evolution with host resilience. In age-aware lung cancer care, constructing such a model requires three core components. First, it must incorporate multimodal inputs, including tumor genomics, longitudinal imaging, treatment history, laboratory trajectories (such as inflammatory markers), comorbidity and frailty measures, and, when feasible, immune profiling (101). Second, it should employ hybrid modeling approaches that combine mechanistic components, such as tumor growth and response dynamics, pharmacokinetics/pharmacodynamics, and toxicity constraints, with machine-learning layers capable of capturing complex, non-linear interactions. Third, the model must be continuously updated as new imaging data, clinical symptoms, and adverse events accrue during therapy. Validation of digital twin models should proceed in a stepwise manner, beginning with internal calibration of predicted versus observed response and toxicity, followed by external validation across institutions and temporal validation in later cohorts to ensure robustness as standards of care evolve (102). Importantly, model performance should be evaluated using clinically actionable endpoints, including the probability of durable response, risk of pneumonitis or interstitial lung disease, hospitalization, functional decline, and competing-risk mortality, rather than relying on discrimination metrics alone. In practice, digital twins can function as a decision sandbox, allowing comparison of plausible strategies such as targeted monotherapy versus targeted therapy followed by immunotherapy, or the addition of microenvironment-modifying agents in biologically older patients (103).

Combining these tools with frailty metrics, wearable health monitors, and advanced imaging will create continuously updated treatment plans that respond dynamically to tumor evolution and changes in patient physiology. Realizing this vision will require a redesign of clinical trials to reflect the complexity of both lung cancer and aging biology. Adaptive trial frameworks that stratify participants by biological age, immune function, and molecular signatures, rather than rigid chronological cutoffs, will allow more representative data collection and equitable access to novel therapies. Lessons from pediatric oncology initiatives like iTHER and ZERO Childhood Cancer demonstrate how global collaboration and precision-based trial design can improve outcomes in rare cancers, and similar approaches are now emerging in geriatric oncology consortia to better serve older patients who have historically been underrepresented in clinical research.

Collectively, these strategies support a shift toward age-aware lung cancer care that integrates biological profiling, AI modeling, and cellular engineering. Although they hold promise for improving survival and quality of life across the lifespan, although many of them, including senolytics and immune rejuvenation, remain investigational and require lung cancer-specific prospective validation.

## 7 Discussion

The consequences of host aging, including immunosenescence, systemic inflammaging, and tissue senescence, represent biologically meaningful variables that can influence lung cancer evolution and treatment response, although their relative contribution varies across patients and clinical contexts. While tumor-centric precision oncology has transformed outcomes in molecularly selected subsets, its translation across the age spectrum remains inconsistent. Current evidence supports an association between aging biology and therapy outcomes, but definitive prospective validation remains limited. Immunosenescence, inflammaging, and lung tissue remodeling should therefore be viewed not as universally deterministic factors, but as interacting modifiers that may constrain therapeutic durability and increase toxicity risk in susceptible individuals.

One central hypothesis is that tumor-centric personalization without concurrent host-directed adaptation is inherently self-limiting. Targeted therapies may successfully engage their molecular targets, but their outcomes are capped by the capacity of an aged immune system and a fibrotic, immunologically restrictive microenvironment to sustain antitumor pressure. The logical extension of this idea is that future regimens must pair tumor-directed interventions with host-targeted co-therapies. Senolytic drugs, inflammasome inhibitors, mitochondrial fitness restorers, and stromal remodeling agents represent not adjunctive strategies but rational partners for targeted therapy and immunotherapy, particularly in biologically older patients. Clinical trials designed with these considerations should move beyond radiographic endpoints to include measures of immune reinvigoration, stromal remodeling, and functional preservation, thereby aligning therapeutic success with both tumor and host adaptation. Pediatric and geriatric lung cancer patients, though often seen as occupying opposite ends of the spectrum, face a strikingly parallel challenge: systemic neglect in clinical research. Pediatric cases are sidelined due to rarity, while older patients are routinely excluded based on comorbidities and organ function thresholds. Both scenarios lead to a deficit of high-quality evidence precisely where it is most needed. This observation suggests that trial infrastructures designed to accommodate low-prevalence cohorts, such as adaptive basket and umbrella studies, should serve both pediatric and geriatric populations. By building shared platforms that unify rare molecular subsets with vulnerable age-defined groups, oncology could transcend the dichotomy between children and the elderly, establishing a continuum of care that is truly inclusive and biology-driven. Biological age emerges as the critical stratifier to replace crude chronological thresholds in both trial design and clinical practice. Tools such as PhenoAgeAccel, epigenetic clocks, telomere length, and frailty indices capture dimensions of aging that more accurately predict therapy tolerance, toxicity, and benefit. A biologically “young” 75-year-old may derive substantial benefit from aggressive targeted and immune combinations, while a biologically “old” 60-year-old might require a more conservative approach emphasizing microenvironmental conditioning and reduced-toxicity regimens. Integrating biological age into randomization schemas and treatment sequencing not only enhances scientific rigor but also promises more equitable outcomes by aligning intervention intensity with physiological resilience rather than demographic categories. The remodeling of the tumor microenvironment represents another axis where age-conscious adaptation can shift outcomes. In elderly patients, ECM stiffening, fibroblast activation, and aberrant integrin and YAP-TAZ signaling create mechanical and biochemical barriers that exclude immune cells and blunt drug penetration. Reframing these processes as therapeutic targets positions microenvironmental remodeling as a geriatric-specific precision strategy. Agents that modulate collagen crosslinking, inhibit integrins, or reprogram activated fibroblasts could act as sequencing primers, administered to restore immune permissiveness before immunotherapy or targeted therapy. The incorporation of spatial-omics and imaging-based biomarkers of tissue stiffness and fibrosis into clinical research could accelerate translation of such strategies into practice, providing tangible tools for tailoring care in elderly populations.

Advances in cellular engineering offer yet another route to counteract the constraints of immune aging. CRISPR-edited and iPSC-derived immune products optimized for checkpoint resistance, metabolic resilience, and persistence in fibrotic tissues may transform immunologically “cold” tumors into responsive ones. Beyond ex vivo engineering, *in vivo* rejuvenation of immune cells through partial reprogramming or targeted epigenetic editing represents a bold but increasingly feasible strategy to restore T-cell diversity and function without increasing oncogenic risk. These innovations illustrate that age-induced immune dysfunction need not be passively accepted but can itself become a therapeutic target, opening new frontiers for durable immunotherapy responses in older adults. The operationalization of such complex, age-aware personalization will likely depend on artificial intelligence and digital twin models. These systems, capable of integrating tumor genomics, biological age biomarkers, wearable-derived data, and comorbidity profiles, can simulate therapeutic strategies and predict both efficacy and toxicity in near real time. By continuously updating predictions as tumors evolve and patients’ physiological states shift, digital twins could transform personalization from a reactive to a predictive and adaptive enterprise. Their integration into prospective trials, with endpoints including not only survival but also quality-adjusted life years and preservation of functional independence, would mark a decisive step toward holistic age-conscious oncology.

Another underexplored dimension of age-informed precision oncology is the biology of early-onset cancers, including the rising incidence of lung and colorectal cancers in adults under 50. While these cases are often linked to hereditary syndromes, emerging data suggest that accelerated biological aging, manifested through early epigenetic drift, telomere attrition, and chronic inflammation, may contribute even in the absence of germline predisposition. Comparing immune and stromal profiles between early- and late-onset lung adenocarcinoma could reveal whether “premature inflammaging” or subclinical immune senescence predisposes younger individuals to disease. Such age-matched comparative research remains logistically challenging but conceptually vital, as it could clarify how biological aging processes begin decoupled from chronological time and reshape risk, progression, and therapy response across the lifespan.

Taken together, these arguments converge on a critical conclusion: age is not a barrier to personalization but a variable to be leveraged. True precision in lung cancer therapy requires a dual focus, targeting tumor biology while simultaneously adapting to the evolving biology of the host. By embedding biological age into trial eligibility, stratification, and therapeutic sequencing, and by developing host-directed co-therapies, cellular engineering approaches, and AI-driven decision support, oncology can transcend the current ceiling of tumor-centric personalization. The future of lung cancer care will depend on dismantling the artificial divide between tumor and host, reframing therapy as a dynamic co-optimization of both systems. Such a transformation promises not only to extend survival but also to safeguard function and quality of life, delivering genuinely durable and equitable outcomes across the lifespan.

Despite growing interest, the clinical application of biological aging in lung cancer remains constrained by several unresolved challenges. Most data linking immunosenescence or aging biomarkers to treatment outcomes are retrospective or correlative. Few trials stratify patients by biological age, and no randomized studies have demonstrated that age-guided treatment modification improves survival or quality of life. Additionally, biomarker thresholds lack standardization, and the interplay between tumor genomics and host aging biology remains incompletely defined. These gaps highlight the need for prospective, biomarker-integrated trial designs before age-aware precision oncology can be implemented at scale.
